# Effects of Rhizosphere Microbial Communities on Cucumber *Fusarium* wilt Disease Suppression

**DOI:** 10.3390/microorganisms11061576

**Published:** 2023-06-14

**Authors:** Fan Yang, Huayan Jiang, Gaozheng Chang, Shen Liang, Kai Ma, Yuxin Cai, Baoming Tian, Xuanjie Shi

**Affiliations:** 1 Institute of Horticulture, Henan Academy of Agricultural Sciences, Graduate T&R Base of Zhengzhou University, Zhengzhou 450002, China; xiaoyuefuxiang@163.com (F.Y.); jhy7327@163.com (H.J.);; 2 School of Agricultural Sciences, Zhengzhou University, Zhengzhou 450001, China

**Keywords:** cucumber *Fusarium* wilt, *Fusarium oxysporum* f. sp. *cucumerinum*, high-throughput sequencing, rhizosphere microbial community

## Abstract

Cucumber *Fusarium* wilt is a worldwide soil-borne disease that seriously restricts the yield and quality of cucumber. The rhizosphere soil microbiome, as the first line of defense against pathogens invading plant roots, plays a key role in rhizosphere immune formation and function. The purpose of this study was to reveal the key microecological factors and dominant microbial flora affecting cucumber resistance and susceptibility to *Fusarium* wilt by analyzing the physical and chemical properties and microbial flora of rhizosphere soil with different degrees of susceptibility and resistance to cucumber *Fusarium* wilt, thereby laying a foundation to establish cucumber resistance to the *Fusarium* wilt rhizosphere core microbiome. Firstly, Illumina Miseq sequencing technology was used to evaluate the physical and chemical properties and microbial groups of cucumber rhizosphere soil at different health levels, and the key environmental factors and microbial factors related to cucumber *Fusarium* wilt were screened out. Subsequently, PICRUSt2 and FUNGuild were used to predict the functions of rhizosphere bacteria and fungi. Combined with functional analysis, the possible interactions among soil physical and chemical properties, cucumber rhizosphere microorganisms, and *Fusarium* wilt were summarized. The results showed that the available potassium content in the rhizosphere soil of healthy cucumber decreased by 10.37% and 0.56%, respectively, compared with the rhizosphere soil of severely susceptible cucumber and mildly susceptible cucumber. Exchangeable calcium content increased by 25.55% and 5.39%; the α diversity Chao1 index of bacteria and fungi in the rhizosphere soil of healthy cucumber was significantly lower than that in the rhizosphere soil of seriously infected cucumber, and the MBC content of its physical and chemical properties was also significantly lower than that in the rhizosphere soil of seriously infected cucumber. There was no significant difference in the Shannon and Simpson diversity indexes between healthy cucumber rhizosphere soil and seriously infected cucumber rhizosphere soil. The results of the β diversity analysis showed that the bacterial and fungal community structure of healthy cucumber rhizosphere soil was significantly different from that of severely and mildly infected cucumber rhizosphere soil. At the genus level, through statistical analysis, LEfSe analysis, and RDA analysis, the key bacterial and fungal genera with potential biomarker values were screened out as *SHA_26*, *Subgroup_22*, *MND1*, *Aeromicrobium*, *TM7a*, *Pseudorhodoplanes*, *Kocuria*, *Chaetomium*, *Fusarium*, *Olpidium,* and *Scopulariopsis*, respectively. The bacteria *SHA_26*, *Subgroup_22,* and *MND1* related to cucumber *Fusarium* wilt inhibition belong to Chloroflexi, Acidobacteriota, and Proteobacteria, respectively. *Chaetomiacea* belongs to Sordariomycates. The results of functional prediction showed that changes to the KEGG (Kyoto Encyclopedia of Genes and Genomes) pathway in the bacterial microbiota were concentrated in tetracycline biosynthesis, selenocompound metabolism, lipopolysaccharide biosynthesis, etc., which were mainly involved in the metabolism of terpenoids and polyketides, energy metabolism, metabolism of other amino acids, glycan biosynthesis and metabolism, lipid metabolism, cell growth and death, transcription, metabolism of cofactors and vitamins, and biosynthesis of other secondary metabolites. The difference in fungi was mainly dung saprotroph–ectomycorrhizal–soil saprotroph–wood saprotroph. Through the correlation analysis and functional predictions of the key environmental factors, microbial flora, and cucumber health index in cucumber rhizosphere soil, we determined that the inhibition of cucumber *Fusarium* wilt was a synergistic effect of environmental factors and microbial flora, and a model diagram was drawn to briefly explain its mechanism. This work will provide a basis for the biological control of cucumber *Fusarium* wilt in the future.

## 1. Introduction

Cucumber *Fusarium* wilt is a worldwide soil-borne disease that occurs in both facilities and open fields, seriously restricting cucumber production and quality. Cucumber *Fusarium* wilt is extremely destructive. It was reported that the disease may be aggravated by climate warming [1]. Cucumber *Fusarium* wilt is caused by *Fusarium oxysporum* f. sp. *cucumerinum* (FOC) [2]. This pathogen can produce chlamydospores, which are difficult to remove once they colonize the soil [3], enabling this pathogen to survive in the soil for many years. This disease has become an important factor restricting the development of modern agriculture. Chemical control is the most economical and effective control method at present, but excessive use of chemicals will seriously disturb rhizosphere microorganisms, make the ecological environment system more fragile, and lead to loss of agricultural biodiversity, which will yield environmental pollution, endanger human and animal health, and also cause “3R” problems such as pathogen resistance, pesticide residue, and pest resurgence. Therefore, the search for new efficient biological pesticides to replace chemical pesticides has become a current focus area of research in the field.

The prevention and control of cucumber *Fusarium* wilt from the perspective of soil microecological balance should be the future development direction. Existing studies have shown that as a core area for plant–soil–microorganism interactions, the rhizosphere is the only vector for soil-borne pathogens to invade plant roots, and rhizosphere microorganisms are considered to be the first line of defense against pathogen invasion [4,5,6]. Rhizosphere microorganisms are, in general, all microorganisms that are tightly attached to a few millimeters of soil on the surfaces of plant roots. These microorganisms are mainly composed of bacteria and represent one of the most active areas of microorganisms in the soil system [7]. To a certain extent, the relationship between rhizosphere microorganisms determines whether pathogenic bacteria can successfully invade plants [8]. KWAK et al. [9] found that compared to susceptible tomato varieties, resistant varieties could obviously recruit *Flavobacterium* to enrich the rhizosphere and used metagenomic sequencing and selective medium separation techniques to cultivate *Flavobacterium TRM1*. The strain was inoculated into the root of susceptible tomato, which significantly improved the resistance of the tomato to *Fusarium* wilt. By comparing the physical and chemical properties and microbial communities in the rhizosphere soil of healthy watermelon, watermelon infected with *Fusarium oxysporum*, and dead watermelon, Meng et al. [10] found that rhizosphere soil NH^4+^, NO^3−^, and hemicellulose can be used as indicators of plant health. At the same time, the relative abundance of potential antagonistic microorganisms in the rhizosphere soil of healthy plants was found to be the highest, while the relative abundance of potential plant pathogens was the lowest. This special microbial composition may be an important factor inhibiting the expression of *Fusarium* wilt disease and impacting plant health. Other studies have shown that the soil with inhibitory effects on strawberry fusarium wilt has beneficial microflora and active secondary metabolism [11]. Shen et al. [12] found that the *Pseudomonas* and *Tumebacillus* genera were enriched in healthy soil. In addition, SHI et al. [13] studied the relationship between the occurrence of potato scab and the composition and function of microbial communities in potato topsoil, speculating that *Stenotrophomonas*, *Agrobacterium*, *Sphingobium*, and *Streptomyces* may aggravate the incidence of scab, while *Geobacillus*, *Curtobacterium*, and *unclassified Geodermatophilaceae* may slow down the incidence of scab. Therefore, it is crucial to determine the resistance of rhizosphere microorganisms to pathogen invasion by analyzing the composition of rhizosphere microorganisms, the degree of correlation between microorganisms, and the types and functions of related antagonistic microorganisms. However, it remains unclear whether there are differences in the composition and diversity of rhizosphere bacteria between resistant and susceptible cucumber varieties and whether there is a correlation between bacterial differences and cucumber resistance to *Fusarium* wilt. With the continuous improvement and development of DNA sequencing technology, it is possible to conduct more comprehensive and accurate research on plant rhizosphere microorganisms. In addition, various algorithms based on 16S rRNA gene sequence assignment can be used to identify biological indicators in disease-suppressed soils.

It is unclear how specific microorganisms in the cucumber rhizosphere soil affect plant health and the development of soil-borne diseases. Therefore, the focus of this research was to detect the wilt-resistant soil of natural cucumbers and study the relationship between its disease inhibition characteristics and rhizosphere soil microorganisms. Our objectives were (1) to study the differences between the soil bacteria and pathogenic bacteria; (2) to identify potential key microbial groups, such as antagonistic microorganisms and pathogenic microorganisms; (3) to analyze the differences in microbial functions between disease-inhibiting soil and pathogenic soil; and (4) determine the microbial indicators related to plant health. This study examined the microbiota of a long-term monocrop cucumber field in Anyang, Henan province, China. Illumina Miseq sequencing technology was used to analyze the rhizosphere microbial community of healthy, mildly infected, and severely infected cucumbers. The key microbial groups involved in the inhibition of cucumber *Fusarium* wilt were identified, and the functional abundance of soil-inhibiting and pathogenic microorganisms was predicted based on gene sequence analysis. To determine the relationship between soil physicochemical properties and key microbial groups, the soil properties were analyzed. Finally, the relationship between major microbial groups and plant health was reviewed. The effect of soil microorganisms on the inhibition of cucumber *Fusarium* wilt pathogen was discussed to provide reference for the prevention and control of cucumber *Fusarium* wilt.

## 2. Materials and Methods

### 2.1. Basic Information on Sampling Sites

In May 2022, the incidence of cucumber *Fusarium* wilt was investigated in the cucumber planting area of Jingdian Town, Neihuang County, Anyang City, Henan Province, and areas matching the following conditions were selected as sampling sites: (1) the presence of a cucumber crop for more than 10 years; (2) a high incidence of more than 45% severe infection with cucumber wilt in soil; (3) 10% incidence of continuous crop cucumber infected with wilt in soil; (4) 5 % or lower, and the distance between the three sampling sites was close (less than 10 km in a straight line). According to the above conditions, Jingdian Town, Neihuang County, Anyang City, Henan Province (112° E, 34° N) was selected as the sampling site. The specific sampling site information is shown in Table 1.

### 2.2. Rhizosphere Soil Sampling

Samples were collected on 20 May 2022. To obtain cucumber rhizosphere soil, cucumber plants were removed and shaken vigorously. The soil that was tightly bound to the root systems was rinsed with sterile salt solution, and centrifugation of the suspension was performed for 10 min at 10,000× *g*, resulting in rhizosphere soil pellets [14]. Each cucumber plant’s rhizosphere soil was mixed as one replicate, resulting in four replicates at each sampling site. The health index (HI) of cucumber was rated as “2” for healthy plants, “1” for lightly affected plants, and “0” for heavily affected plants. After soil samples were collected, the samples were stored in ice packs and transported within 12 h to the laboratory. A portion of the rhizosphere soil samples was stored at −80 °C until DNA extraction, and a portion was immediately used to perform physicochemical analysis.

### 2.3. Determination of Soil Chemical Properties and Extraction of Soil DNA

To assess soil conditions in the study area, three technical replicates were conducted to determine the physicochemical properties of HR, R, and S rhizosphere soils. Soil chemical properties, including soil pH, soil electrical conductivity (EC), available nitrogen content (AN), available phosphorus content (AP), available potassium content (AK), available iron content (AFe), exchangeable calcium content (ECa), available copper content (ACu), dehydrogenase activity (S-DHA), invertase activity (S-SR), urease activity (S-UR), and microbial biomass carbon (MBC) were determined according to the above method [15]. A DNeasy Power Soil Kit (QIAGEN GmbH, Hilden, Germany) was used for soil genomic DNA extraction. A NanoDrop spectrophotometer (Thermo Scientific, Wilmington, DE, USA) was used to determine the concentration and purity of the extracted DNA, and a 1% agarose gel was used to assess the DNA quality. For subsequent analysis, the isolated DNA was stored at −20 °C.

### 2.4. Tag Sequencing of Bacterial and Fungal Communities

Sequencing libraries for bacteria and fungi were constructed as described previously [16,17]. The study of bacterial and fungal communities was based on the sequencing of 16S rRNA genes and ribosomal DNA ITS region-end amplicons using the Illumina MiSeq platform of Personal Biotechnology Ltd. (Shanghai, China). The bacterial 16S rRNA gene fragment was amplified using bacterial primers 338F (5′-ACTCCTACGGGGAGGCAGCAG-3′) and 806R (5′-GGACTACHVGGGTWTCTAAT-3′) for the V3-V4 hypervariable region [18]. The ITS region was targeted at primers 1737F (5′-GGAAGTAAAAGTCGTAACAAGG-3′) and 2043R (5′-GCTGCGTTCTTCATCGATGC-3′) [19]. PCR reaction conditions were as follows: pre-denaturation at 95 °C for 3 min, 95 °C for 30 s, 55 °C for 30 s, 72 °C 40 s, 35 cycles for fungi or 27 cycles for bacteria, AOA (ammonia-oxidizing archaea) and AOB (ammonia-oxidizing bacteria), and a final extension at 72 °C for 10 min and then at 10 °C until halted by user. In the PCR for the fungal sequences, 5 min were spent at 95 °C, followed by 35 cycles of 45 s at 94 °C, 50 s at 58 °C, 30 s at 68 °C, and a final extension of 10 min at 65 °C. An AxyPrep Gel Extraction Kit (Axygen, Union City, CA, USA) was used to recover PCR products from 2% agarose gels. DNA MiSeq was sequenced commercially using an Illumina MiSeq sequencer. The resulting DNA sequencing data were uploaded to the National Center for Biotechnology Information (Bethesda, MD, USA) Sequence Read Archive (SRA) database under BioProject accession numbers PRJNA921884 and PRJNA921900.

### 2.5. Bioinformatics and Statistical Analysis of Sequencing Data

Splitting of ~300 bp raw DNA sequences into each sample was based on unique barcodes and the trimming of adapter and primer sequences in QIIME (version 1.2.0) and USEARCH (version 9.1.13) [20]. After quality control, OTUs were clustered based on 97% pairwise identity, and chimeras were filtered using the UPARSE algorithm [21]. Further removal of mitochondrial and nonbacterial OTUs and OTUs with relative abundance below 0.01% allowed us to classify representative sequences for each OTU using the Ribosomal Database Project classifier (RDP, version 11.5). The affiliations were classified using the RDP bacterial 16S database or the UNITE fungal ITS database [22].

The relative abundance of a given classification group for each sample was calculated by dividing the number of sequences belonging to the group by the total number of sequences. We first calculated the alpha diversity. Using alpha_rarefaction.py, the OTU table was diluted to read up to 80,985 readings. This value was used because it represents the lowest sequencing depth obtained from the sample. In order to calculate beta diversity, the unrefined “filtered_OTUtable” was first normalized with the R package metagenomeSeq (version 1.12). Currently, available sequencing technologies suffer from bias due to uneven sequencing depth, so we use cumulative and scaling (CSS) to avoid this problem [23]. Bray–Curtis and weighted and unweighted Unifrac phase difference matrices were calculated using standardized OTU tables, and principal coordinate analysis (PCoA) was performed using the Phyloseq package (v.1.10) to explore differences in microbial communities [24]. QIIME calculated the weighted UniFrac distance unweighted group averaging (UPGMA) clusters [25,26] to represent beta diversity. UPGMA clustering is a hierarchical clustering method using average links that can be employed to interpret a distance matrix. The relative abundance at the phylum level, family level, and genus level was calculated using OTU based on histograms generated for each taxonomic level using the Origin 9.9.0.225 software to compare the distribution of microorganisms in disease-suppressing and disease-causing soils. Using the Galaxy online interface, linear discriminant analysis (LDA), effect size (LEfSe), and genetic analysis were performed. Genes with an LD score of 3.5 were considered significant biomarkers for each treatment [27]. We also use redundancy analysis to test the relationships between microbial community structure and soil environmental variables with vegan package in the R program. We predicted the potential functions of bacterial communities using PICRUSt2 (systematic genetic Investigation of Communities by Reconstruction of observed States 2) in the KEGG pathway database [28]. According to the previous method [29], the FUNGuild (Fungi Functional Guild) V1.0 [30] online platform was used to classify the ecological functions of fungi. The correlation between soil physicochemical properties, key bacteria and fungi, and plant HI was analyzed using the Origin 9.9.0.225 software. The Krona 2.7.1 software was used to visualize the taxonomic composition of the samples.

### 2.6. Statistical Analyses

All statistical analyses were performed using IBM SPSS 20.0 (IBM Corporation, New York, NY, USA) and R software (Version 3.5.2). To analyze the effects of *Fusarium* wilt on soil properties and enzyme activity, we performed a one-way analysis of variance (ANOVA) and post hoc multiple comparison test (Tukey HSD). Kruskal–Wallis tests were used to determine the significance of microbial alpha diversity. As a statistical significance test, the Wilcoxon rank sum test was used for a comparison of the two groups. All statistical tests performed in this study were considered significant at *p* < 0.05, while *p* < 0.01 was considered extremely significant.

## 3. Results

### 3.1. Comparison of Physical and Chemical Properties of HR, R, and S

Twelve properties of rhizosphere soil were assessed in each group, including EC, AN, AP, AK, AFe, ECa, ACu, pH, S-DHA, S-SR, S-UR, and MBC, to determine the relationship between chemical properties, enzyme activities, and cucumber *Fusarium* wilt incidence. According to the results, HR, R, and S soil enzyme activities changed significantly in both physicochemical and enzyme activity properties (Table S1). The contents of AK, AN, AP, and AFe in S were significantly higher than those in HR and R, which increased by 9.39%, 8.89%, 12.00%, 20.71%, 27.12%, 37.48%, 19.54%, and 29.19%, respectively. However, the contents of ACu and ECa in S significantly decreased by 14.17%, 29.02%, 25.55%, and 21.31%, respectively. The results of soil enzyme activity showed that S-DHA, S-SR, and S-UR in S were significantly higher than those of the other two soils, which indirectly indicated that soil microbial biomass or energy exchange in S was higher than HR and R. Compared with HR and R, the MBC value of S increased by 22.45% and 38.78%, and the EC value increased by 39.91% and 41.28%, respectively, indicating that cucumber *Fusarium* wilt was related to the rhizosphere soil microbial biomass and soil EC value. There was no significant difference in the rhizosphere soil pH of cucumber under three different health conditions (*p* > 0.05), indicating that the occurrence of cucumber *Fusarium* wilt did not affect the rhizosphere soil pH. In conclusion, the results of soil physical and chemical properties showed that soil nutrient content, soil enzyme activity, and microbial community composition were all related to the occurrence of cucumber *Fusarium* wilt.

### 3.2. Differences in the Composition of Microbial Communities in HR, R, and S

**Figure 1:**
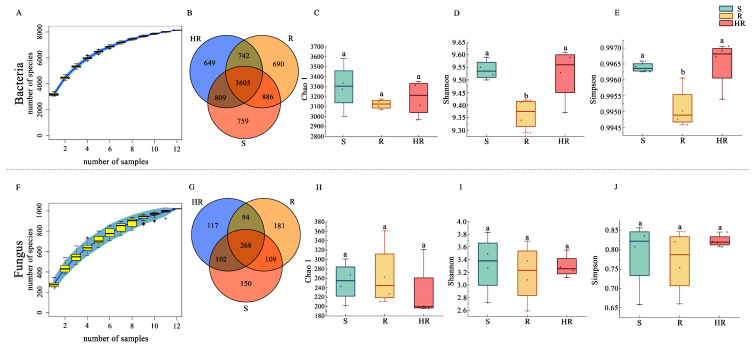
Alpha diversity of rhizosphere microbial communities. The species accumulation curves of bacteria (**A**) and fungi (**F**). Venn diagrams were created for bacteria (**B**) and fungi (**G**) identified in the three groups. Box plots show the variation of Chao1 index (**C**,**H**), Shannon index (**D**,**I**), and Simpson index (**E**,**J**) for bacteria and fungi from the three groups. Different letters within the graph indicate significant differences between the means (*p* < 0.05).

An Illumina MiSeq sequencer was used to sequence 16S rRNA gene amplicons and ITS gene amplicons. We plotted the species accumulation curves of the bacterial and fungal community sequencing results to determine whether the sample size of this study was sufficient and estimated community richness. The results showed that when the number of samples exceeded 10, the number of identified new species basically reached the plateau, and the accumulation curve of bacteria tended to be flatter than that of fungi. In this study, 12 samples were sufficient to reflect the species composition of the community. The results also showed that the number of identified bacterial species was much higher than that of fungi, possibly because more bacteria than fungi were identified in the database. In order to study the common and unique species among the three samples, we plotted the Venn diagram. For the bacterial community, 8140 OTUs were generated (Figure 1B). Additionally, 1021 OTUs were generated for the fungal community (Figure 1G) and the α-diversity indices were calculated using QIIME2. The Chao1 index represented the richness of the microbial community, and the Shannon index and Simpson index represented the diversity of the microbial community. In the bacterial community, the Chao1 index of S (3299.79) was higher than that of HR and R (3188.32, 3126.10) (Table S2), but the difference was not significant (Figure 1C). In the comparison of the Shannon diversity index and Simpson diversity index, there was no significant difference in bacterial diversity between S and HR (Figure 1D,E), and the bacterial diversity of the two groups was significantly higher than that of the R group. In the fungal community, there were some differences in the Chao1 index (253.23, 265.34, 229.16), Shannon index (3.33, 3.18, 3.30), and Simpson index (0.79, 0.77, 0.82) of S, R, and HR, but there was no significant difference (Figure 1H–J).

**Figure 2:**
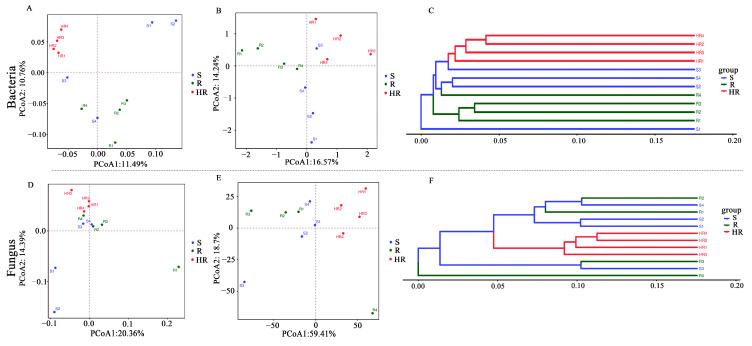
Beta diversity of the rhizosphere microbial community. Beta diversity of bacterial (**A**,**B**) and fungal (**D**,**E**) communities was represented using principal coordinate analysis (PCoA) using weighted and unweighted unifrac. UPGMA clustering analysis based on Bray–Curtis for bacterial (**C**) and fungal (**F**) communities.

A PCoA analysis was performed using weighted unifrac and unweighted unifrac bacterial and fungal OTUs based on the combined sequences of 16S rRNA genes and ITS fragments in soil samples to visualize the similarities and dissimilarities between microbial communities (Figure 2A,B,D,E). UPGMA cluster analysis based on a sample distance matrix was used to show the similarities between samples (Figure 2C,F). Using weighted unifrac-based PCoA analysis (Figure 2A,D), only the microbial abundance in the sample was considered. The results showed that the bacterial community of HR could be distinguished from R and S, while R and S could not. The fungal abundance in the three sample groups was similar. Using PCoA analysis based on unweighted unifrac analysis (Figure 2B,E), only the microbial structure in the sample was considered, enabling the bacterial and fungal structures among HR, R, and S to be distinguished. This result indicates that there were differences in the bacterial and fungal community structures among HR, R, and S. UPGMA cluster analysis showed that the bacterial and fungal community structures were not similar between HR and R and S (Figure 2C,F). However, the fungal community structures of R and S were similar. The results of the α and β diversity analysis showed that the microbial community structure of HR was different from that of R and S.

### 3.3. Screening of Key Bacteria and Fungi in HR, R, and S

**Figure 3:**
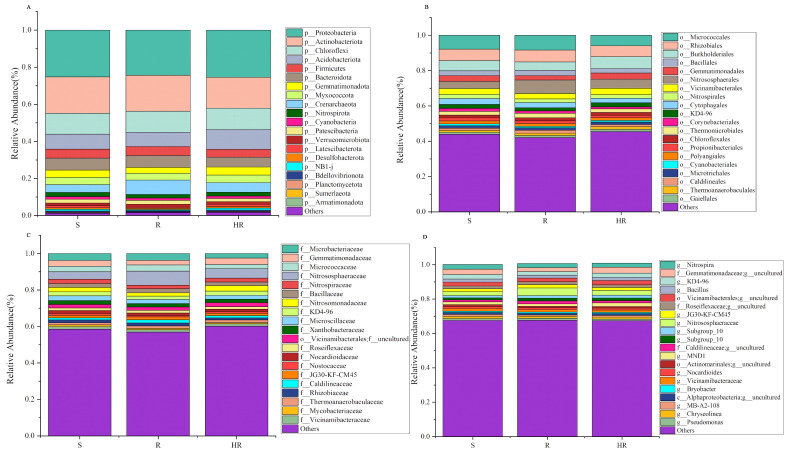
A histogram (relative abundance) of bacterial community composition at the phylum (**A**), order (**B**), family (**C**), and genus (**D**) levels (top 20).

**Figure 4:**
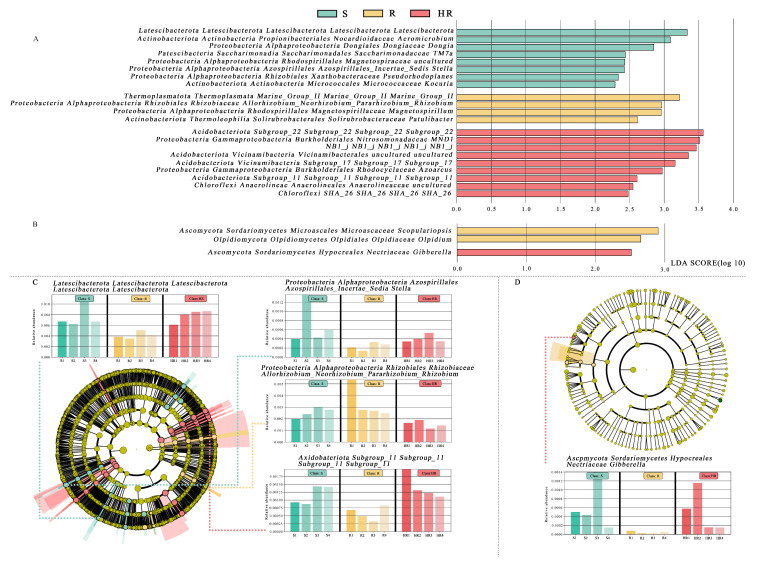
The linear discriminant analysis effect value (LEfSe) of bacterial (**A**) and fungal (**B**) communities was greater than 2.0 when the P value was less than 0.05. The histogram showed the LDA score of microbial groups. Groups that meet the LDA significance threshold >2.0 are displayed. The taxonomic cladogram showed the main bacterial (**C**) and fungal (**D**) taxa in the sample community from phylum to genus (from inside to outside). The size of the node corresponds to the average relative abundance of the classification unit; hollow nodes represent groups with no significant difference between groups, while nodes of other colors indicate that these groups show significant differences between groups, and the groups represented by colors are more abundant in the sample. The histogram showed the relative abundance of bacteria (**C**) and fungi (**D**) in the three sample groups.

All bacterial OTUs were assigned to 50 bacterial phyla. Specifically, 13 bacterial phyla had an average relative abundance of more than 1%, and, together, these phyla accounted for more than 94% of the total sequences recovered. In order, the five most abundant bacterial phyla were Proteobacteria (24.96%), Actinobacteriota (18.60%), Chloroflexi (11.36%), Acidobacteriota (8.82%), and Bacteroidota (6.08%). The relative abundance of Actinobacteriota in HR (16.65%) was lower than that of R (19.43%) and S (19.73%), while the relative abundance of Acidobacteriota (10.64%) was higher than that of R (7.68%) and S (8.14%) (Figure 3A). At the order level, the relative abundance of Burkholderiales in HR (6.67%) was higher than that in R (4.84%) and S (5.72%) (Figure 3B). At the family level, the relative abundance of Nitrosomonadacea was higher in HR (5.17%) and R (7.77%) than in S (4.12%), and the relative abundance of Microbacterium was higher in S (3.81%) than in HR (2.47%) and R (3.75%) (Figure 3C). At the genus level, the relative abundance of *MND1* in HR (2.16%) was higher than that in R (1.34%) and S (1.55%) (Figure 3D). The LEfSe analysis method (Figure 4A) revealed 67 significantly different species among S, R, and HR. A total of 23 bacterial species were screened as dominant in S, with 11 bacterial genera in R and 33 bacterial genera in HR. At the genus level, we observed the relative abundance of the *Latescibacterota* of Latescibacterota (LDA = 3.33, *p* = 0.024), the *Aeromicrobium* of Actinobacteria (LDA = 3.09, *p* = 0.021), the *Dongia* of Proteobacteria (LDA = 2.85, *p* = 0.018), the *TM7a* of Patescibacteria (LDA = 2.44, *p* = 0.021), uncultured Proteobacteria (LDA = 2.43, *p* = 0.020), the *Stella* of Proteobacteria (LDA = 2.42, *p* = 0.015). the *Pseudorhodoplanes* of Proteobacteria (LDA = 3.34, *p* = 0.015) and the *Kocuria* of Actinobacteriota (LDA = 2.29, *p* = 0.024) in S were higher than those of HR and R. The relative abundance of the *Marine_Group_II* (LDA = 3.22, *p* = 0.007), the *Allorhizobium_Neorhizobium_Pararhizobium_Rhizobium* of Proteobacteria (LDA = 2.96, *p* = 0.023), the *Magnetospirillum* of Proteobacteria (LDA = 2.95, *p* = 0.005), and the *Patulibacter* of Actinobacteriota (LDA = 2.62, *p* = 0.021) in R were higher than those in the other two soils. In HR, the relative abundance of the *Subgroup_22* of Acidobacteriota (LDA = 3.57, *p* = 0.018), the *MND1* of Proteobacteria (LDA = 3.51, *p* = 0.015), the *NB1_j* of NB1_j (LDA = 3.47, *p* = 0.01), uncultured Acidobacteriota (LDA = 3.35, *p* = 0.01), the *Subgroup_17* of Acidobacteriota (LDA = 3.16, *p* = 0.024), the *Azoarcus* of Proteobacteria (LDA = 2.97, *p* = 0.012), the *Subgroup_11* of Acidobacteriota (LDA = 2.61, *p* = 0.023), uncultured Chloroflexi (LDA = 2.55, *p* = 0.023), and the *SHA_26* of Chloroflexi (LDA = 2.48, *p* = 0.023) was higher than that in the other two soils. Figure 4C,D and Figure S1 show the taxonomic hierarchy of the sample taxa from the phylum to the genus level. The relative abundance of genera with significant differences between groups in the three groups was analyzed. The results showed that although the *Latescibacterota* of Latescibacterota was the dominant bacteria in S in the LEfSe analysis, it only showed the highest relative abundance in S3; these bacteria presented lower abundance in other sample groups of S, even lower than the relative abundance in HR (Figure 4C). Similarly, the relative abundance of the Stella of Proteobacteria was only highest in S2 (Figure 4C). These two prominent bacterial genera in S were not statistically significant. The relative abundance of the dominant bacteria *Allorhizobium_Neorhizobium_Pararhizobium_Rhizobium* of Proteobacteria in R was only the highest in R1 (Figure 4C), so it was not statistically significant. The *Subgroup_11* of Acidobacteriota in HR was also not statistically significant (Figure 4C), so it was not involved in the subsequent analysis.

**Figure 5:**
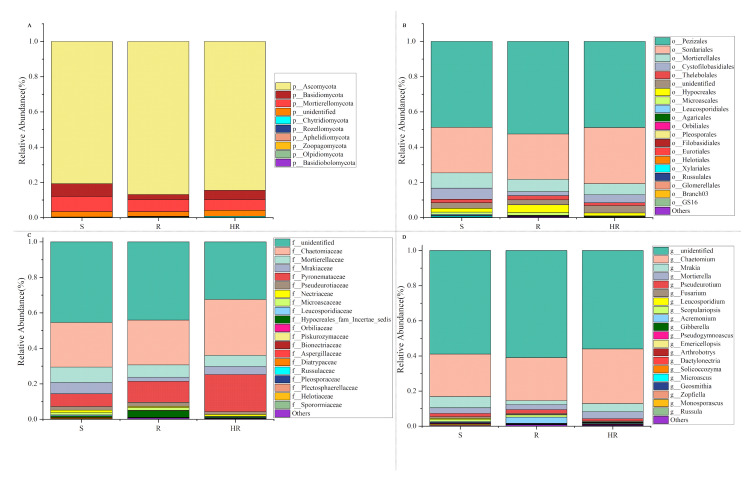
A histogram (relative abundance) of fungal community composition at the phylum (**A**), order (**B**), family (**C**), and genus (**D**) levels (top 20).

Compared with rhizosphere bacteria, the fungal community diversity was lower. All fungal OTUs belonged to 10 fungal phyla. Among them, Ascomycota had the highest relative abundance, accounting for more than 84% of the total recovered sequences. At the phyla level, the relative abundance of Ascomycota in HR (84.46%) was higher than that in S (80.75%). The relative abundance of Mortierellomycota (8.58%) and Basidiomycota (7.23%) in S was higher than that in HR (6.20%, 5.16%) and R (6.98%, 2.80%) (Figure 5A). At the order level, the relative abundance of Sordariales was higher in HR (31.84%) than in R (25.75%) and S (25.85%), and the relative abundance of Mortierellales was higher in S (8.62%) than in HR (6.22%) and R (6.94%) (Figure 5B). At the family level, the relative abundance of Chaetomiaceae in HR (31.47%) was higher than that in R (25.29%) and S (25.18%); the relative abundance of Pyronemataceae in HR (20.90%) was higher than that in R (11.80%) and S (7.11%); and the relative abundance of Mortierellaceae in S (8.70%) was higher than that in HR (6.24%) and R (6.97%) (Figure 5C). At the genus level, the relative abundance of *Chaetomium* was higher in HR (31.12%) than in R (24.42%) and S (24.11%), while the relative abundance of *Fusarium* was highest in S (1.05%) and lowest in HR (0.54%). The relative abundance of *Scopulariopsis* was higher in R (1.21%) and S (1.10%) than in HR (0.23%). *Monosporascus* was only present in S (Figure 5D). The LEfSe analysis method (Figure 4B) showed that there were no dominant fungi in S. There were four dominant fungi in R and one dominant fungus in HR. At the genus level, the *Scopulariopsis* of Ascomycota (LDA = 2.96, *p* = 0.025) and the *Olpidium* of Olpidiomycota (LDA = 2.613, *p* = 0.023) were the dominant bacteria in R. The relative abundance of the *Gibberella* of Ascomycota (LDA = 2.42, *p* = 0.024) in HR was higher than that in the other two soils. However, its relative abundance was only higher in HR3, so it was not statistically significant (Figure 4D).

### 3.4. Correlation of Dominant Genera of Microbial Communities with Plant HI and Environmental Factors

**Figure 6:**
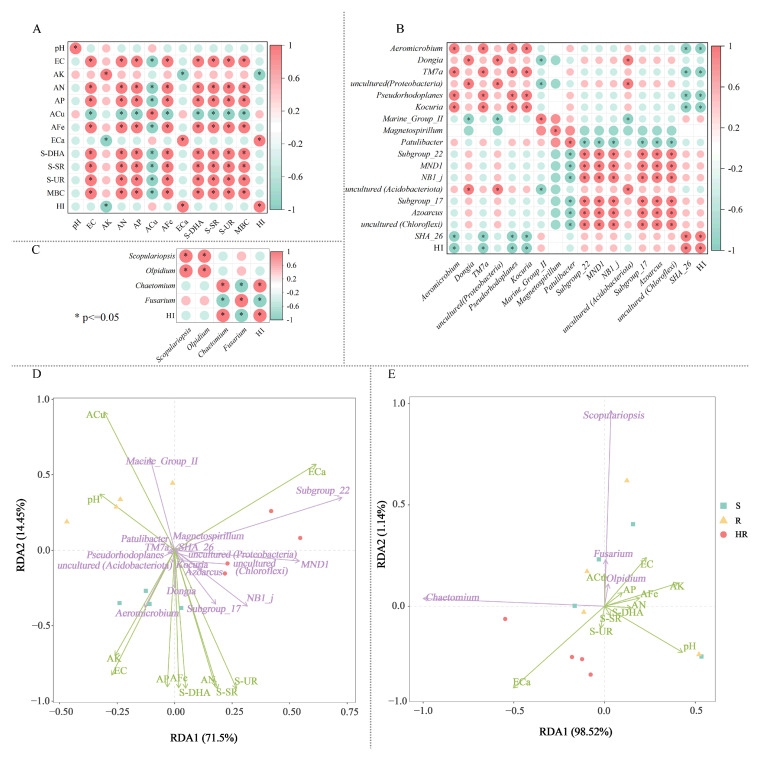
The correlation analysis heatmap of soil factors of HR, R, and S with screened bacteria (**A**) fungi (**B**), key bacteria, and fungi (**C**). Redundancy analysis (RDA) of the relationship between soil bacterial (**D**) and fungal (**E**) community structure and soil factors under different groups. In the correlation analysis, the legend shows the correlation coefficient value, red represents the positive correlation, blue represents the negative correlation; color depth indicates the strength of the correlation; * *p* < 0.05. In RDA analysis, the green arrows represent key bacteria and fungi at the genus level, whereas soil factors are shown with red arrows. Correlation between soil factors (ACu, AFe, ECa, EC, OM, MBC, S-DHA, S-UR, S-SR) and RDA axes are shown by both length and angle of arrows.

In order to better understand the causes of microbial community changes, we performed a Spearman correlation analysis on the relationship between microbial community composition and soil physical and chemical properties (Figure 6). Spearman correlation analysis showed that soil ECa content was positively correlated with the relative abundance of plant HI and negatively correlated with AK (Figure 6A). Plant HI was significantly positively correlated with the relative abundance of *SHA_26*, and significantly negatively correlated with the relative abundance of *Aeromicrobium*, *TM7a*, *Pseudorhodoplanes*, and *Kocuria* (Figure 6B). Plant HI was positively correlated with the relative abundance of *Chaetomium* and negatively correlated with *Fusarium* (Figure 6C). RDA analysis between the soil physicochemical properties and key bacteria and fungi showed that there was a positive correlation between ECa, *Subgroup_22*, and *MND1* (Figure 6D,E). Aeromierobium had a strong positive correlation with AK and EC, respectively. ACu had a strong positive correlation with *Fusarium*, *Olpidium*, and *Scopulariopsis*. There was also a positive correlation between plant HI and the relative abundance of *Burkholderiales* and *MND1* (Figure 6B). Plant HI was significantly and positively correlated with the relative abundance of *Chaetomium* and negatively correlated with *Fusarium* (Figure 6C). Two diagrams were drawn according to the results of the correlation analysis (Figure 6D,E). The above results showed a direct correlation between soil factors and microbial changes, which also indicated that these key rhizosphere bacteria and fungi may have certain synergistic and antagonistic effects in cucumber rhizosphere soil.

### 3.5. Analysis of Microbial Community Function of HR, R, and S

**Figure 7:**
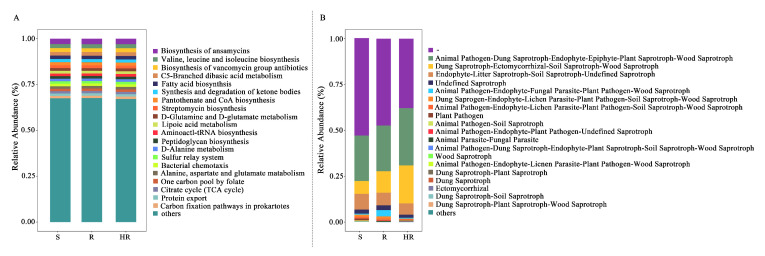
Picrust2 (**A**) and FUNGulid (**B**) annotated and predicted functions based on the bacterial and fungal community composition of HR, R, and S.

To investigate the potential changes in soil microorganisms in the three sample groups, we compared the functional abundance of bacterial and fungal communities based on a sequence analysis of 16S rRNA genes’ ITS markers. PICRUSt2 and FUNGulid were applied to predict microbial functions, thereby providing a basis for understanding the microbial community itself and its potential interactions with the host cucumber. By carefully studying the KEGG homologs in microbial metabolism (Figure 7A), the results showed that in the three-level prediction, the highest relative abundance of the KEGG pathway was in the biosynthesis of ansamycins, valine, leucine, and isoleucine biosynthesis, biosynthesis of vancomycin group antibiotics, C5-branched dibasic acid metabolism, and fatty acid biosynthesis. In the level 2 predictions, the overall relative abundance of carbohydrate metabolism, amino acid metabolism, and metabolism of cofactors and vitamins was higher. In the level 1 prediction, metabolism accounted for most of the whole. Then, we used the STAMP 2.1.3 software to analyze the differences in KEGG pathways among the three groups (Figure S2). The results showed that the difference between HR and R and S was greatest, while that between R and S was smaller. Therefore, we focus on analyzing the functional prediction results between HR and R and between HR and S. A total of 67 pathways between HR and R showed significant differences (*p* < 0.05), in which six pathways showed extremely significant differences (*p* < 0.01). Lipopolysaccharide biosynthesis, tetracycline biosynthesis, and photosynthesis were enriched in HR, while tyrosine metabolism, biosynthesis of siderophore group nonribosomal peptides, and linoleic acid metabolism were significantly present in R. A total of 42 pathways between HR and S showed significant differences (*p* < 0.05), in which 5 pathways showed extremely significant differences (*p* < 0.01). Tetracycline biosynthesis and selenocompound metabolism were extremely significantly enriched in HR, and Xylene degradation, primary bile acid biosynthesis, and bisphenol degradation were enriched in S.

FUNGulid is a database of functional annotations of fungi that currently covers over 12,000 fungi. Fungulid was used to predict fungal function by annotating against the structural composition of fungal communities (Figure 7B). We used STAMP 2.1.3 software to analyze the functional prediction results between HR and R and HR and S (Figure S3). The results showed that the functional abundance of animal pathogen–plant pathogen–undefined saprotroph in R was significantly higher than that in HR (*p* = 0.05). The functional abundance of dung saprotroph–ectomycorrhizal–soil saprotroph–wood saprotroph was significantly higher than that of S (*p* = 0.013), which was more than three times that of HR.

### 3.6. Possible Mechanism of Rhizosphere Microorganisms Inhibiting Cucumber Fusarium Wilt

According to the correlation among soil physicochemical properties, microbial community, and plant HI in Figure 7, combined with RDA analysis between key bacteria and fungi and soil physical and chemical properties, we selected soil ECa content and AK content as key environmental factors. Bacteria *SHA_26*, *Subgroup_22*, *MND1*, *Aeromicrobium*, *TM7a*, *Pseudorhodoplanes* and *Kocuria*, fungi *Chaetomium*, *Fusarium*, *Olpidium,* and *Scopulariopsis* were the key microbial factors. The key factors related to cucumber *Fusarium* wilt inhibition were soil ECa content, *SHA_26*, *Subgroup_22*, *MND1*, and *Chaetomium*. To predict the functions of bacterial and fungal communities, we determined the relative functional abundance of tetracycline biosynthesis, selenocompound metabolism, lipopolysaccharide biosynthesis, cell cycle–Caulobacter, biosynthesis of vancomycin group antibiotics, fatty acid biosynthesis, polyketide sugar unit biosynthesis, pantothenate and CoA biosynthesis, indole alkaloid biosynthesis, RNA polymerase, carbon fixation pathways in prokaryotes, oxidative phosphorylation, and pantothenate and CoA biosynthesis in HR, which was significantly higher than that in R and S. The effects of rhizosphere bacteria and fungi on FOC may occur through the following four pathways: (1) competitive nutrition and spatial location with FOC; (2) toxin production of FOC; (3) changes to plant root exudates affecting the growth of FOC; and (4) microbe-associated molecular patterns (MAMPs) produced by the interaction between microorganisms and hosts activating detection of the plant immune system.

## 4. Discussion

The cucumber is one of the world’s top ten vegetables and an important cash crop in China, cultivated throughout the country in all seasons of the year. At present, soil-borne diseases are the most common and serious threat to cucumber production facilities. Cucumber wilt is a destructive soil-borne disease caused by *Fusarium oxysporum* f. sp. *cucumerinum*, leading to significant damage to crop yield and quality and causing significant economic losses globally. Thus, studying rhizosphere soil physicochemical properties and the composition of cucumber microbial communities is important for the biological control of cucumber wilt.

### 4.1. Relationship between Rhizosphere Soil Physicochemical Properties and Plant Health

It is well known that abiotic soil factors contribute to the development of plant diseases. Soil physicochemical properties and enzyme activities have important effects on microbial activity. In this study, we determined whether there were significant differences in rhizosphere soil pH among three healthy cucumber plants and found no significant relationship between soil pH and the incidence of cucumber *Fusarium* wilt. Many studies have shown that the incidence of *Fusarium* wilt is low in high pH soils [31]. However, some studies have shown that the disease severity score decreases with a decrease in acidic pH [32,33], with no correlation between the severity of plant disease and pH [34]. It can be seen that the relationship between soil pH and plant disease occurrence may be affected by the plant and soil types studied. EC values that are too high can form reverse osmotic pressure, leaving the root system dry and brown and making the roots unable to absorb water and nutrition. According to Nam et al. [35], strawberry *Fusarium* wilt severity increases with an increase in EC values. According to some studies, salinity promotes the growth of *V. dahliae* in the root and shoot, which leads to an increase in pepper verticillium wilt severity [36,37,38]. It was suggested by Aviles et al. [39] that EC plays a key role in promoting the growth of Verticillium wilt in accordance with the findings of this study.

The results of the soil chemical property analysis showed that the contents of ACu and ECa in the rhizosphere soil of healthy and wilt-infected cucumber plants were significantly higher than those in severely wilted plants, whereas AK, AP, AN, and AFe contents were significantly lower than those in severely wilted plants. However, only ECa and AK were significantly correlated with the plant health index. This result indicates that the rhizosphere soil of severe *Fusarium* wilt plants lacks ECa nutrients and is rich in AK. In addition to improving plant biofilms, ECa can enhance plant resistance to environmental stress and improve the selective absorption capacity of plant cell biofilms. As a chemotactic agent of some important enzymes, ECa enhances the activity of α-amylase and phospholipase. Many plant diseases are closely related to calcium content. Jiang et al. [40] demonstrated that increasing the calcium concentration within a certain range can increase the peroxidase activity of tomato plants so as to better control tomato bacterial wilt. ECa may have the ability to control cucumber *Fusarium* wilt. Generally, the rhizosphere soils of susceptible plants contain lower levels of AK, AN, and AP [41,42,43,44,45]. Interestingly, in this study, the AK, AN, and AP contents of the rhizosphere soil of severely diseased plants were higher than those of the other two soils. There may also be a correlation between growth restriction and nutrient uptake by plants [46,47]. Further research is necessary to examine the relationship between these three factors and *Fusarium* wilt. The presence of AFe in the environment is conducive to the formation of chlorophyll and the metabolic process of nitrogen. Soluble organic matter such as root exudates, soil organic matter, and microbial activity metabolites complexed or chelated with AFe in the soil solution help to increase the iron concentration in the soil solution and prompt iron to move to the root system. According to this study, the amount of iron available in the rhizosphere soil of severely diseased plants was significantly higher than that in the other two soils, likely due to a high level of microbial activity. An imbalance of trace elements will yield a change in the microbial population structure of the soil and an increase in pathogenic microbes. In a study by He et al. [48], researchers examined the changes in trace elements in continuous cropping cucumber cultivars and found that effective iron ions increased as continuous cropping years increased and that *Fusarium* number increased linearly as continuous cropping stubbles increased.

Since the secretion of microbial activity in the soil is one of the main sources of soil enzymes, the number of microorganisms has a significant correlation with enzyme activity, but the results of different scholars’ studies are inconsistent, which may be related to factors such as soil type, plant species, maintenance management, and soil microbial community. In this experiment, S-DHA, S-SR, and S-UR were found to be significantly higher in S compared to the other two soils, and Pearson correlation analysis showed that all three were positively correlated with the microbial population. The highest MBC in S indicated that this soil contained the highest total soil microbial population. This result suggests that the highest soil enzyme activity in the rhizosphere soil of severely susceptible cucumber plants may be related to metabolism caused by a high number of microbes.

### 4.2. Key Microbiota Associated with Plant Health 

Soil microorganisms play an important role in the occurrence and prevention of plant diseases [49,50]. It was reported that when attacked by pathogens, plant roots resist pathogens by altering their root exudates [51] to attract beneficial microorganisms and enhance their microbial activity [11]. In terms of microbial diversity, abundance, and activity, Janvier et al. [52] observed that these factors may not be consistent indicators of soil health.

**Figure 8:**
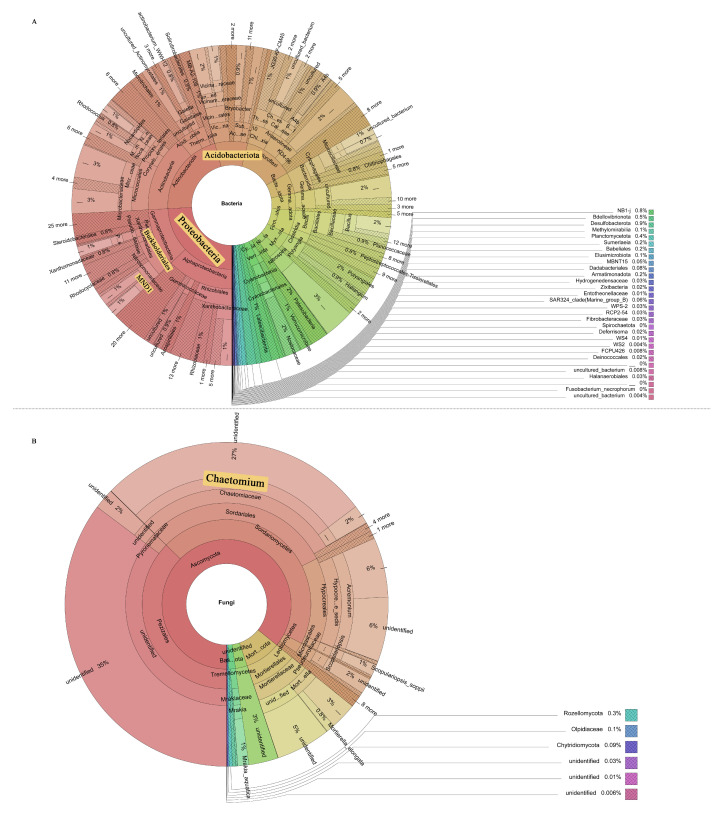
Species composition of bacteria (**A**) and fungi (**B**). The prominent part of the yellow background indicates the key microbial groups.

It is well known that plant pathogens can cause diseases by directly infecting plants. However, it was demonstrated that soil microbial communities play an important role in disease development as they interact directly with plant pathogens and regulate disease development in various ways [53,54]. It was observed that most of the antagonistic bacteria used to control pathogens belong to Proteobacteria [55], while Acidobacteria are usually associated with disease inhibition [56,57]. As shown in this study, Proteobacteria and Acidobacteriota were more abundant in healthy plants’ rhizosphere soil compared to the two other groups. In addition, LEfSe analysis revealed that most of the dominant bacteria in the rhizosphere soil of healthy plants belonged to Acidobacteria and Proteobacteria (Figure 8A). The relative abundance of Chloroflexi *SHA_26* was significantly positively correlated with plant HI. At present, there is no research on *SHA_26*, so the function of the *SHA_26* genus is not understood. Chloroflexi is a type of bacteria that produces energy through photosynthesis. At the order level, the relative abundance of Burkholderiales in healthy plants was found to be higher (Figure 8A). Burkholderiales are members of the Amoebae subfamily that produce secondary metabolites and volatile organic compounds [58,59]. Burkholderiales have the ability to inhibit pathogens. In addition, they are an important plant growth-promoting variety of bacteria in the rhizosphere environment. It was reported that the rhizosphere biological control strain most prevalent in the wheat take-all disease inhibition period is Burkholderiales [60]. Previous studies showed that [52,61,62] Burkholderiales inhibit multiple pathogens in the rhizosphere soil of healthy plants. Burkholderiales may control *Fusarium* wilt in the rhizosphere soil of healthy plants. At the family level, Nitrosomonadaceae are the dominant bacteria in the rhizosphere soil of healthy plants. Nitrosomonadaceae are used for the bioremediation of toxic chemicals in the soil. The addition of Nitrosomonadaceae reduces the loss of nitrogen and the time needed to stabilize the nitrogen profile. Li et al. [62] reported that Nitrosomonadaceae were significantly and positively correlated with Panax ginseng yield. Additionally, the relative abundance of the *MND1* genus in the rhizosphere soil of healthy plants was higher than that in the other two groups. It is well known that the presence of many members of the *MND1* genus can improve soil quality. As emphasized by Sun et al. [63], *MND1* plays an important role in the mineralization of organic phosphorus and the dissolution of inorganic phosphorus. *MND1* belongs to the Nitrosomonadaceae family and is an ammonia-oxidizing bacterium involved in nitrogen fixation, ammonia oxidation, and other nitrogen cycle processes [64]. However, in this experiment, the contents of AN and AP in healthy soil were lower than those in R and S, so *MND1*’s involvement in inhibiting cucumber *Fusarium* wilt may be related to the other functions of this genus. Wang et al. [65] showed that *MND1* may be valuable for promoting the degradation of BP-3 in plant growth.

The relative abundance of *Chaetomium* accounted for a large proportion of all three groups, and we found a positive correlation between their relative abundance and HI values. *Chaetomium* belongs to the Sordariales of Sordariomycetes (Figure 8B). Members of the genus *Chaetomium* are widely distributed in the soil and can produce a large amount of cellulase and physiologically active secondary metabolites, which can effectively degrade cellulose, lignin, and other difficult-to-degrade macromolecular organic matter, as well as antagonize some microorganisms in the soil, thereby inhibiting the growth of pathogenic bacteria [66,67]. Li et al. [68] reported that some compounds isolated from *Chaetomium* showed significant inhibitory effects on the pathogenic fungus of Panax notoginseng root rot. The abundance of pathogens is a direct factor affecting plant health [69]. In this study, we found that the relative abundance of *Fusarium* was negatively correlated with HI values. In addition to *Fusarium*, several other fungi associated with plant pathogenicity, such as *Monosporascus*, *Leucosporidium*, and *Dactylonectri*, had the highest relative abundance in the rhizosphere soil of severely affected plants. *Monosporascus cannonballus* is a common pathogen causing the root rot of melon, which we found to be present only in the rhizosphere soil of severely damaged plants. A functional prediction by FUNGulid found that the rhizosphere soils of severely affected plants had the highest functional abundances of dung saprotroph, ectomycorrhizal saprotroph, soil saprotroph, and wood saprotroph. This result may be directly related to these plant pathogens.

**Figure 9:**
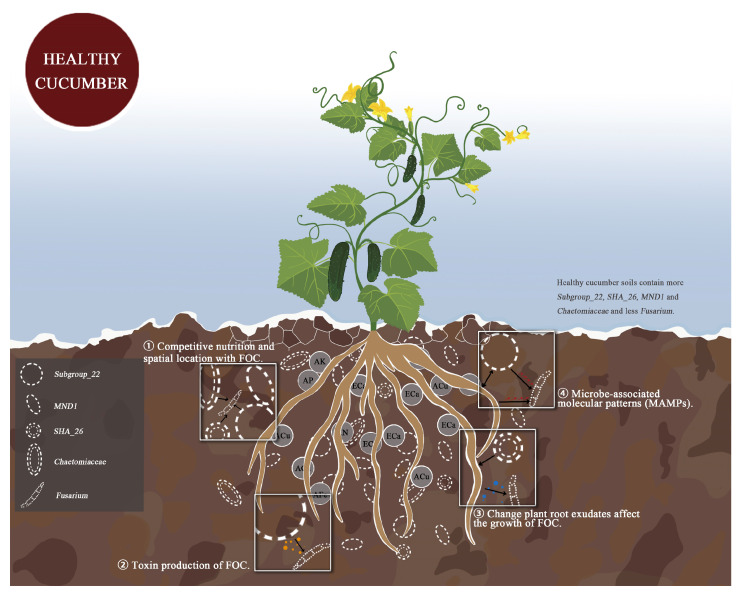
Rhizosphere soil bacteria and fungi combined with soil factors through four possible ways synergistic inhibition of cucumber Fusarium wilt possible mechanism diagram.

Plants with a healthy rhizosphere, lightly afflicted plants, and severely afflicted plants differ significantly in their rhizosphere soil microbial community composition. In maintaining plant health, the rhizosphere microbiota plays a key role [61]. Some evidence indicates that microbial communities differ between suppressed soils and favorable soils, with suppressed soils showing a greater abundance of taxa associated with disease suppression [14]. The microbiome of the rhizosphere of healthy plants may, therefore, be able to control soil pathogens such as wilt. In this study, Acidobacteriota *Subgroup_22*, Chloroflexi *SHA_26*, and *MND1* were significantly enriched in the rhizosphere soil of healthy plants. Many studies showed that Acidobacteriota are associated with a variety of diseases. At present, there is no experimental evidence demonstrating that Chloroflexi *SHA_26* and *MND1* have a preventive effect on cucumber *Fusarium* wilt, but they are known to be beneficial to plant growth. *Chaetomium*, which has an inhibitory effect on pathogenic bacteria, is also abundant in the rhizospheres of healthy plants. However, it is not sufficient to interpret these differences as a result of soil–plant interactions. Studies are needed to determine how these microbial and chemical properties directly affect plant health. Finally, we combined correlation analysis to draw a possible mechanism map, revealing that environmental factors, rhizosphere bacteria, and fungi inhibit the occurrence of *Fusarium* wilt through four possible pathways (Figure 9). Whether the specific microbial communities and soil factors in the rhizosphere soil of healthy cucumbers have the ability to inhibit disease remains to be determined in future studies.

## 5. Conclusions

Despite using the same growth conditions for cucumber, there were significant differences observed in soil physical and chemical properties and microbial communities under the three different health conditions. ECa levels in the rhizosphere soil of healthy and susceptible plants may be able to control soil-borne diseases. In the rhizosphere soil of healthy cucumber plants, Acidobacteriota *Subgroup_22*, Chloroflexi *SHA_26*, *MND1,* and *Chaetomium* as pathogen antagonists presented the highest relative abundance, while the relative abundance of potential plant pathogens was the lowest. This result may be due to the fact that this special microbial composition inhibits the expression of *Fusarium* wilt and the development of healthy plants.

In summary, in the rhizosphere soil, we detected ECa, bacteria Acidobacteriota *Subgroup_22*, Chloroflexi *SHA_26*, *MND1*, and fungi *Chaetomiacea* were associated with *Fusarium* wilt inhibition. Ultimately, we conclude that the physicochemical properties and microbial community composition of cucumber rhizosphere soil in the three different health states can be used to judge plant health.

## Figures and Tables

**Table 1:** Basic information on sampling points.

Site	Test Abbreviation (Abbr)	Longitude (E)	Latitude (N)	Altitude (m)	Soil Type
Cucumber *Fusarium* wilt disease serious place	S	112.7611	34.6649	131	loam soil
Cucumber *Fusarium* wilt disease light place	R	112.7613	34.6653	131	loam soil
Places where cucumber wilt does not occur	HR	112.7615	34.6639	131	loam soil

## Data Availability

All raw sequence data have been made available in the NCBI Sequence Read Archive (SRA) database under the bioproject accession number PRJNA921884 and PRJNA921900.

## Supplementary Materials

The following supporting information can be downloaded at: https://www.mdpi.com/article/10.3390/microorganisms11061576/s1, Table S1: Soil physical and chemical properties of HR, R and S; Table S2: Bacterial alpha diversity index table; Table S3: Fungal alpha diversity index table; Figure S1: The taxo-nomic cladogram showed the main bacterial (A) and fungal (B) taxa in the sample community from phylum to genus (from inside to outside). The size of the node corresponds to the average relative abundance of the classification unit; hollow nodes represent groups with no significant difference between groups, while nodes of other colors indicate that these groups show significant differences between groups, and the groups represented by colors are more abundant in the sample. The histogram showed the relative abundance of bacteria (A) and fungi (B) in the three sample groups; Figure S2: Prediction and comparison of rhizosphere bacterial community functions. The changes of bacterial functional group composition were inferred by PICRUSt2. STAMP software was used to analyze the difference of KEGG function between HR and R (A) and HR and S (B), and Welch’s two-sided t-test and Bonferroni multiple test correction method was used. The abscissa of the left column graph represents the average value of a certain function percentage, the ordinate represents the function name, and different colors represent different groups. The figure on the right represents the proportion of species abundance differences within the set confidence interval; Figure S3. Prediction and comparison of rhizosphere fungal community functions. The changes of bacterial functional group composition were inferred by FUNGuild. STAMP software was used to analyze the difference of KEGG function between HR and R (A) and HR and S (B), and Welch’s two-sided t-test and Bonferroni multiple test correction method was used. The abscissa of the left column graph represents the average value of a certain function percentage, the ordinate represents the function name, and different colors represent different groups. The figure on the right represents the proportion of species abundance differences within the set confidence interval.
