# Supplementary material for: Effects of Rhizosphere Microbial Communities on Cucumber Fusarium wilt Disease Suppression
Source: Microorganisms. 2023 Jun 14;11(6):1576. doi: 10.3390/microorganisms11061576 (PMC10301182; doi:10.3390/microorganisms11061576)
Supplement: Supplementary file 1 [file microorganisms-11-01576-s001.zip › microorganisms-2339846-supplementary.pdf]

Table S1. Soil physical and chemical properties of HR, R and S.

Sample	EC (us/cm)	AN (mg/kg)	AP (mg/kg)	AK (mg/kg)	AFe (mg/kg)	ECa (cmol/kg)	ACu (ppm)	pH	S-DHA (U/g)	S-SR (U/g)	S-UR (U/g)	MBC (g/kg)
S	439.00±15.30a	138.79±3.98a	15.91±0.18a	468.89±22.39a	20.75±0.69a	6.39±0.02c	8.69±0.14c	7.78±0.02a	2.90±0.11a	18.23±0.07a	79.52±0.60a	0.49±0.03a
R	257.25±2.99b	109.74±2.09c	9.89±0.49c	418.59±20.23b	14.77±0.44c	6.85±0.03a	12.18±0.11a	7.80±0.01a	1.35±0.16c	14.38±0.15c	61.48±0.86c	0.30±0.03c
HR	262.25±7.93b	122.82±4.07b	11.71±0.37b	414.98±22.65b	16.71±0.26b	7.23±0.05b	10.03±0.34b	7.77±0.01a	1.90±0.11b	16.28±0.10b	72.11±0.96b	0.38±0.03b

* Soil physical and chemical properties of S, R and HR. Values are means ± standard error (SE), n=3. Different letters in the columns indicate significant differences between means ($p<0.05$).

Table S2. Bacterial alpha diversity index table.

Sample	Chao1	Shannon	Simpson
S	3299.79±205.84	9.54±0.03	0.10±0.00
R	3126.10±41.39	9.37±0.05	0.10±0.00
HR	3188.32±154.86	9.52±0.09	0.10±0.00

* Bacterial alpha diversity index table. The value is mean ± standard error (SE), n = 3.

Table S3. Fungal alpha diversity index table.

Sample	Chao1	Shannon	Simpson
S	253.23±36.21	3.33±0.41	0.79±0.08
R	265.34±58.30	3.18±0.41	0.77±0.07
HR	229.16±53.14	3.30±0.16	0.82±0.01

* Fungal alpha diversity index table. The value is mean ± standard error (SE), n = 3.

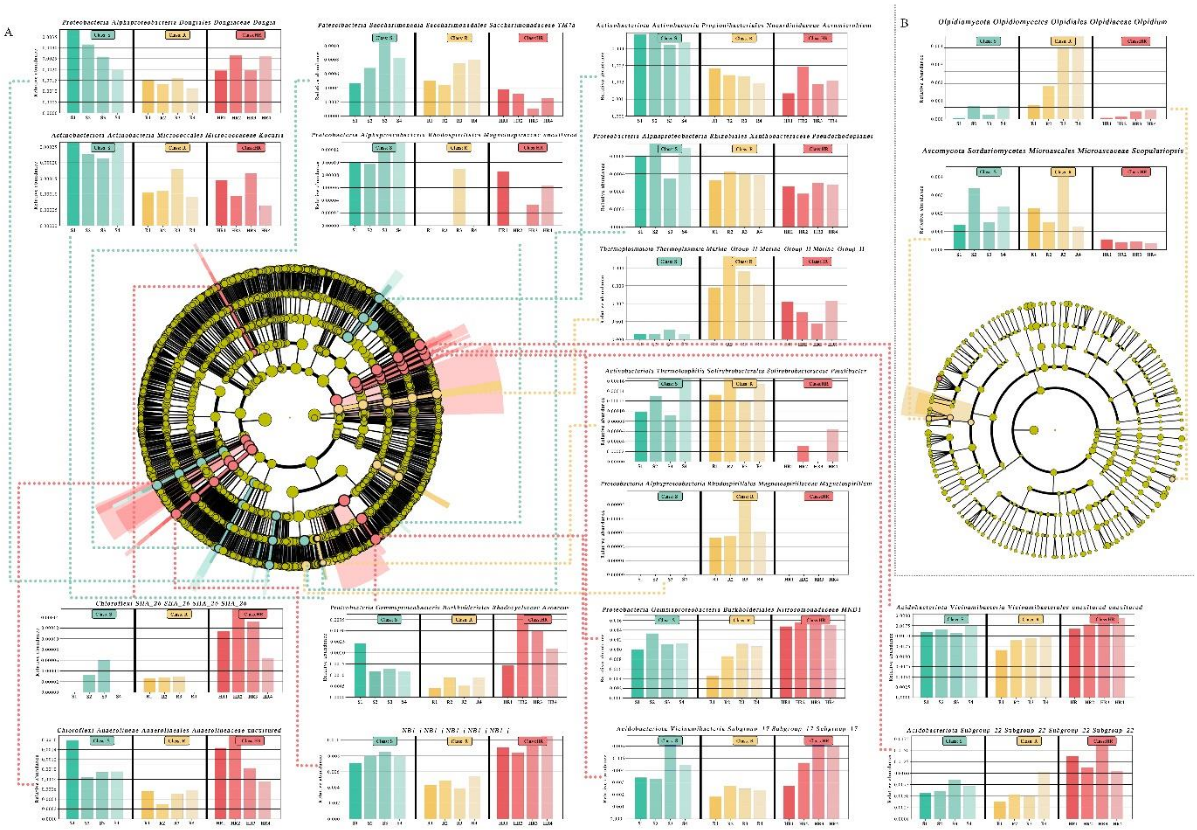

Figure S1. The taxo-nomic cladogram showed the main bacterial (A) and fungal (B) taxa in the sample community from phylum to genus (from inside to outside). The size of the node corresponds to the average relative abundance of the classification unit; hollow nodes represent groups with no significant difference between groups, while nodes of other colors indicate that these groups show significant differences between groups, and the groups represented by colors are more abundant in the sample. The his-togram showed the relative abundance of bacteria (A) and fungi (B) in the three sample groups.

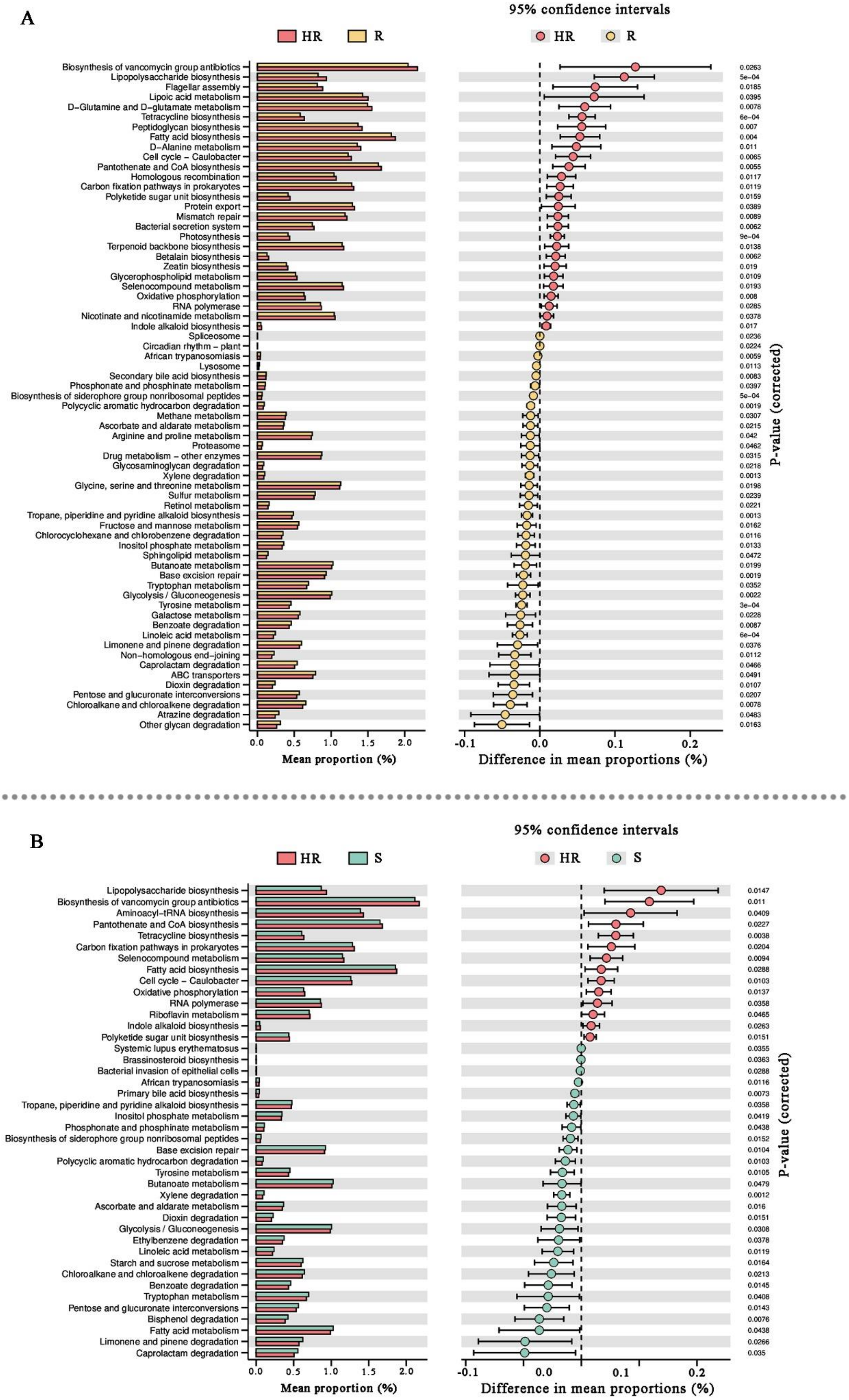
A
95% confidence intervals
HR
R
Biosynthesis of vancomycin group antibiotics
Lipopolysaccharide biosynthesis
Flagellar assembly
Lipoic acid metabolism
D−Glutamine and D−glutamate metabolism
Tetracycline biosynthesis
Peptidoglycan biosynthesis
Fatty acid biosynthesis
D−Alanine metabolism
Cell cycle − Caulobacter
Pantothenate and CoA biosynthesis
Homologous recombination
Carbon fixation pathways in prokaryotes
Polyketide sugar unit biosynthesis
Protein export
Mismatch repair
Bacterial secretion system
Photosynthesis
Terpenoid backbone biosynthesis
Betalain biosynthesis
Zeatin biosynthesis
Glycerophospholipid metabolism
Selenocompound metabolism
Oxidative phosphorylation
RNA polymerase
Nicotinate and nicotinamide metabolism
Indole alkaloid biosynthesis
Spliceosome
Circadian rhythm − plant
African trypanosomiasis
Lysosome
Secondary bile acid biosynthesis
Phosphonate and phosphinate metabolism
Biosynthesis of siderophore group nonribosomal peptides
Polycyclic aromatic hydrocarbon degradation
Methane metabolism
Ascorbate and aldarate metabolism
Arginine and proline metabolism
Proteasome
Drug metabolism − other enzymes
Glycosaminoglycan degradation
Xylene degradation
Glycine, serine and threonine metabolism
Sulfur metabolism
Retinol metabolism
Tropane, piperidine and pyridine alkaloid biosynthesis
Fructose and mannose metabolism
Chlorocyclohexane and chlorobenzene degradation
Inositol phosphate metabolism
Sphingolipid metabolism
Butanoate metabolism
Base excision repair
Tryptophan metabolism
Glycolysis / Gluconeogenesis
Tyrosine metabolism
Galactose metabolism
Benzoate degradation
Linoleic acid metabolism
Limonene and pinene degradation
Non−homologous end−joining
Caprolactam degradation
ABC transporters
Dioxin degradation
Pentose and glucuronate interconversions
Chloroalkane and chloroalkene degradation
Atrazine degradation
Other glycan degradation
0.0 0.5 1.0 1.5 2.0
Mean proportion (%)
-0.1 0.0 0.1 0.2
Difference in mean proportions (%)
0.0263
5e−04
0.0185
0.0395
0.0078
6e−04
0.007
0.004
0.011
0.0065
0.0055
0.0117
0.0119
0.0159
0.0389
0.0089
0.0062
9e−04
0.0138
0.0062
0.019
0.0109
0.0193
0.008
0.0285
0.0378
0.017
0.0236
0.0224
0.0059
0.0113
0.0083
0.0397
5e−04
0.0019
0.0307
0.0215
0.042
0.0462
0.0315
0.0218
0.0013
0.0198
0.0239
0.0221
0.0013
0.0162
0.0116
0.0133
0.0472
0.0199
0.0019
0.0352
0.0022
3e−04
0.0228
0.0087
6e−04
0.0376
0.0112
0.0466
0.0491
0.0107
0.0207
0.0078
0.0483
0.0163
P-value (corrected)
B
95% confidence intervals
HR
S
Lipopolysaccharide biosynthesis
Biosynthesis of vancomycin group antibiotics
Aminoacyl−tRNA biosynthesis
Pantothenate and CoA biosynthesis
Tetracycline biosynthesis
Carbon fixation pathways in prokaryotes
Selenocompound metabolism
Fatty acid biosynthesis
Cell cycle − Caulobacter
Oxidative phosphorylation
RNA polymerase
Riboflavin metabolism
Indole alkaloid biosynthesis
Polyketide sugar unit biosynthesis
Systemic lupus erythematosus
Brassinosteroid biosynthesis
Bacterial invasion of epithelial cells
African trypanosomiasis
Primary bile acid biosynthesis
Tropane, piperidine and pyridine alkaloid biosynthesis
Inositol phosphate metabolism
Phosphonate and phosphinate metabolism
Biosynthesis of siderophore group nonribosomal peptides
Base excision repair
Polycyclic aromatic hydrocarbon degradation
Tyrosine metabolism
Butanoate metabolism
Xylene degradation
Ascorbate and aldarate metabolism
Dioxin degradation
Glycolysis / Gluconeogenesis
Ethylbenzene degradation
Linoleic acid metabolism
Starch and sucrose metabolism
Chloroalkane and chloroalkene degradation
Benzoate degradation
Tryptophan metabolism
Pentose and glucuronate interconversions
Bisphenol degradation
Fatty acid metabolism
Limonene and pinene degradation
Caprolactam degradation
0.0 0.5 1.0 1.5 2.0
Mean proportion (%)
-0.1 0.0 0.1 0.2
Difference in mean proportions (%)
0.0147
0.011
0.0409
0.0227
0.0038
0.0204
0.0094
0.0288
0.0103
0.0137
0.0358
0.0465
0.0263
0.0151
0.0355
0.0363
0.0288
0.0116
0.0073
0.0358
0.0419
0.0438
0.0152
0.0104
0.0103
0.0105
0.0479
0.0012
0.016
0.0151
0.0308
0.0378
0.0119
0.0164
0.0213
0.0145
0.0408
0.0143
0.0076
0.0438
0.0266
0.035
P-value (corrected)

Figure S2. Prediction and comparison of rhizosphere bacterial community functions. The changes of bacterial functional group composition were inferred by PICRUSt2. STAMP software was used to analyze the difference of KEGG function between HR and R (A) and HR and S (B), and Welch 's two-sided t-test and Bonferroni multiple test correction method was used. The abscissa of the left column graph represents the average value of a certain function percentage, the ordinate represents the function name, and different colors represent different groups. The figure on the right represents the proportion of species abundance differences within the set confidence interval.

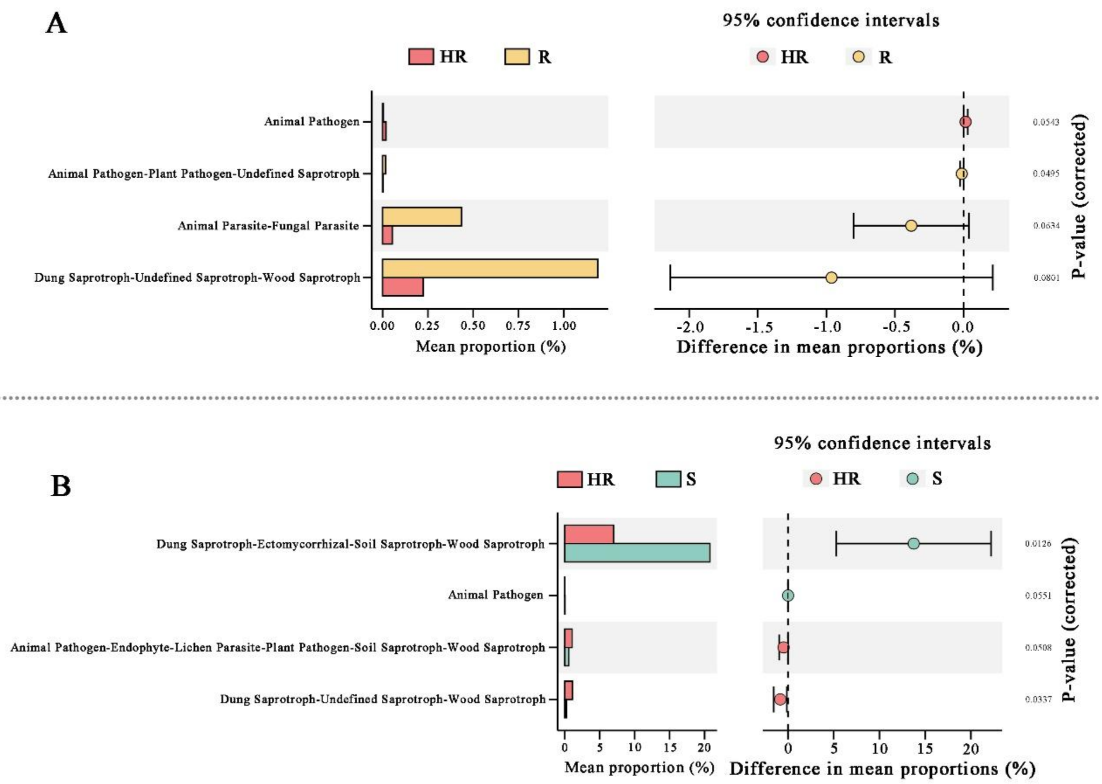

Figure S3. Prediction and comparison of rhizosphere fungal community functions. The changes of bacterial functional group composition were inferred by FUNGuild. STAMP software was used to analyze the difference of KEGG function between HR and R (A) and HR and S (B), and Welch 's two-sided t-test and Bonferroni multiple test correction method was used. The abscissa of the left column graph represents the average value of a certain function percentage, the ordinate represents the function name, and different colors represent different groups. The figure on the right represents the proportion of species abundance differences within the set confidence interval.
